# Induction of autophagy by spermidine is neuroprotective via inhibition of caspase 3-mediated Beclin 1 cleavage

**DOI:** 10.1038/cddis.2017.161

**Published:** 2017-04-06

**Authors:** Yi Yang, Sicong Chen, Yuqing Zhang, Xiaoxia Lin, Yiyin Song, Zhaoliang Xue, Haoran Qian, Shanshan Wang, Guihua Wan, Xiaoxiang Zheng, Lihui Zhang

**Affiliations:** 1 Department of Pharmacology, Hangzhou Key Laboratory of Medical Neurobiology, School of Medicine, Hangzhou Normal University, Hangzhou, China; 2 Department of Biomedical Engineering, Zhejiang Provincial Key Laboratory of Cardio-Cerebral Vascular Detection Technology and Medicinal Effectiveness Appraisal, Zhejiang University, Hangzhou, China; 3 Clinical Research Center, The 2nd Affiliated Hospital, Zhejiang University School of Medicine, Hangzhou, China; 4 Department of Neurosurgery, Sir Run Run Shaw Hospital, Medical College of Zhejiang University, Hangzhou, China; 5 Department of General Surgery, Institute of Micro-Invasive Surgery of Zhejiang University, Sir Run Run Shaw Hospital, Medical College of Zhejiang University, Hangzhou, China

## Abstract

Spermidine, a natural polyamine presented widely in mammalian cells, has been implicated to extend the lifespan of several model organisms by inducing autophagy. However, the effect of spermidine against neuronal damage has not yet been fully determined. In this study, neuronal cell injury was induced by treating PC12 cells and cortical neurons with 1 *μ*M staurosporine (STS). We found that STS-induced cell injury could be efficiently attenuated by pretreatment with 1 mM spermidine. Spermidine inhibited the caspase 3 activation induced by STS. Moreover, STS incubation resulted in autophagic degradation failure, which could be attenuated by the pretreatment of spermidine. Knocking down the expression of Beclin 1 efficiently suppressed autophagosome and autolysosome accumulation, and abolished the protective effects of spermidine against STS-induced neurotoxicity. Increased Beclin 1 cleavage and partial nuclear translocation of Beclin 1 fragment was detected in STS-treated cells, which could be blocked by spermidine, pan-caspase inhibitor or caspase 3-specific inhibitor. The nuclear translocation of Beclin 1 fragment universally occurs in damaged neurons. Beclin 1 mutation at the sites of 146 and 149 prevented the intracellular re-distribution of Beclin 1 induced by STS. In addition, intraperitoneal injection of spermidine ameliorated ischemia/reperfusion-induced neuronal injury in the hippocampus and cortex of rats, possibly via blocking caspase 3 activation and consequent Beclin 1 cleavage. Our findings suggest that caspase 3-mediated Beclin 1 cleavage occurs in acute neuronal cell injury both *in vitro* and *in vivo*. The neuroprotective effect of spermidine may be related to inhibition of the caspase 3-mediated Beclin 1 cleavage and restoration of the Beclin 1-dependent autophagy.

Spermidine, a naturally formed polyamine in mammalian living cells, has crucial roles in various cellular processes under pathophysiological conditions.^1^ Recent studies highlight its ability in extending lifespan of many model organisms, including yeast, nematodes, flies and mice.^2, 3^ Importantly, the longevity-promoting activity of spermidine is known to be associated with its capacity in enhancing autophagy.^2, 4^ Exogenous supply of spermidine ameliorates the age-induced memory impairment in fruit flies by eliciting autophagic activity.^5^ Nevertheless, the underlying mechanism of neuroprotection yielded by spermidine against acute or chronic neuronal injury remains largely unknown.

Autophagy and apoptosis are well-characterized processes that contribute to the maintenance of cellular and tissue homeostasis. The crosstalk between autophagy and apoptosis has been documented in various physiological and pathological conditions; in agreement with this notion, several crucial molecules have been identified as the points of convergence between two pathways.^6, 7^ Beclin 1, the mammalian ortholog of yeast Atg6, is indispensable for the initiation of autophagy.^8^ Emerging lines of evidence suggests that Beclin 1 is a novel substrate of caspases, and can be cleaved by caspases at *DXXD* motifs, where D is aspartate and X represents any amino acid.^9, 10, 11, 12, 13, 14^ After cleavage, Beclin 1 loses its capacity in inducing autophagy, but the C-terminal fragment leads to the release of cytochrome C from mitochondria and therefore triggers apoptosis.^12^ However, the involvement of caspase-mediated Beclin 1 cleavage in neuronal cells has not yet been fully clarified. Considering the crucial role of Beclin 1 in modulation of the interaction between apoptosis and autophagy, we speculate that spermidine may exert a neuroprotective action by regulating the autophagy/apoptosis switch node Beclin 1.

In this study, staurosporine (STS) was adopted to induce acute neuronal injury in cultured PC12 cells and cortical neurons, and the potential neuroprotective effects of spermidine against STS were clarified. In addition, the spermidine-induced neuroprotection was further evaluated in ischemia/reperfusion (I/R)-induced neuronal injury *in vivo*. Our findings indicate that spermidine may represent a promising natural compound for the treatment of neurological diseases.

## Results

### Spermidine prevents STS-induced neuronal cell damage *in vitro*

**Figure 1:**
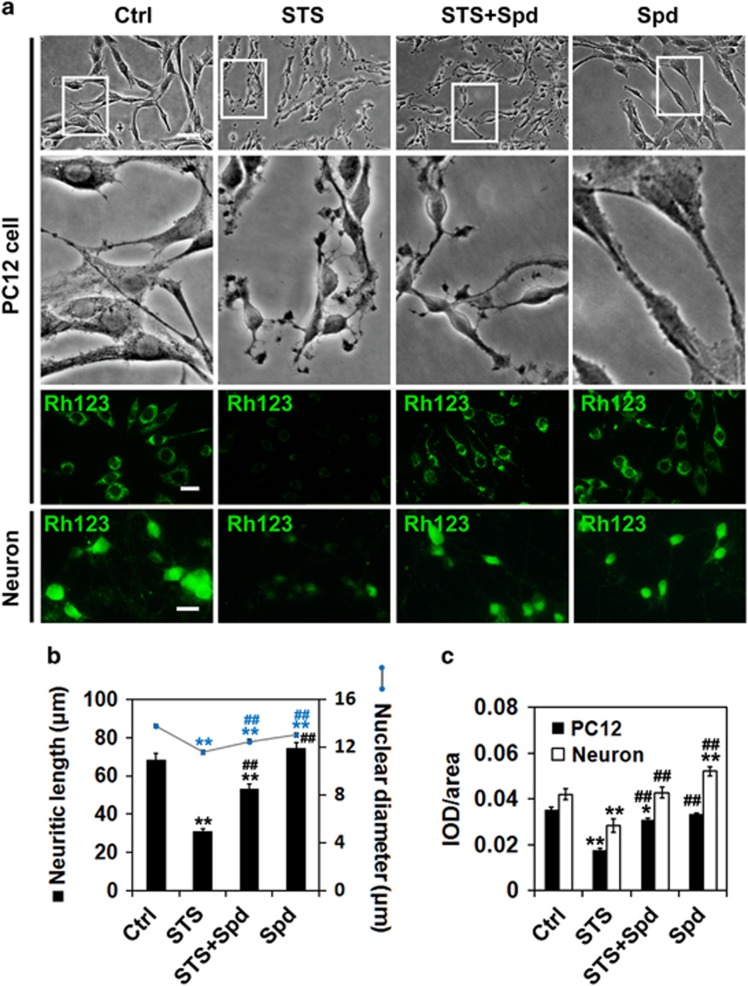
Spermidine prevented STS-induced neuronal cell death. PC12 cells or primary cultured cortical neurons were treated with STS, STS+spermidine (STS+Spd) or Spd. Untreated cells were used as control (Ctrl). (**a**) Morphological alterations of PC12 cells following different treatments were examined under light microscope. Scale bar, 50 *μ*m. Cells were stained with Rhodamine 123 (Rh123) for analyzing the mitochondrial membrane potential. Scale bar, 20 *μ*m. (**b**) The mean neuritic length and nuclear diameter of PC12 cells, was calculated. For each group, 60 cells were utilized for analysis and data were calculated from three independent experiments. The black and blue markers indicate the statistical significance of the data when comparing neuritic length and nuclear diameter, respectively. (**c**) The average IOD per area in Rh123-stained samples was calculated. For each group, 15 images were used for analysis and data were calculated from three independent experiments. *P<0.05, **P<0.01 *versus* Ctrl; ^##^*P*<0.01 *versus* STS

STS, a widely used pro-apoptotic agent, provoked PC12 cell injury *in vitro*. After 1 h of STS (1 *μ*M) incubation, the mean neuritic length and nuclear diameter were both decreased (*P*<0.01 *versus* control) (Figures 1a and b). A profound loss of mitochondrial membrane potential was detected in cells exposed to STS (Figures 1a and c). Pretreatment with spermidine (1 mM) significantly suppressed the neurite shortening, nuclear size reduction and prevented the loss of mitochondrial membrane potential induced by STS treatment (*P*<0.01 *versus* STS) (Figures 1a and c). Moreover, treatment with spermidine alone at the dose of 1 mM for 2 h had no significant influences on cells. Similar to that seen in PC12 cells, spermidine exerted prominent neuroprotection in primary cortical cultures against STS treatment (Figures 1a and c).

### Spermidine inhibits caspase 3 activation

**Figure 2:**
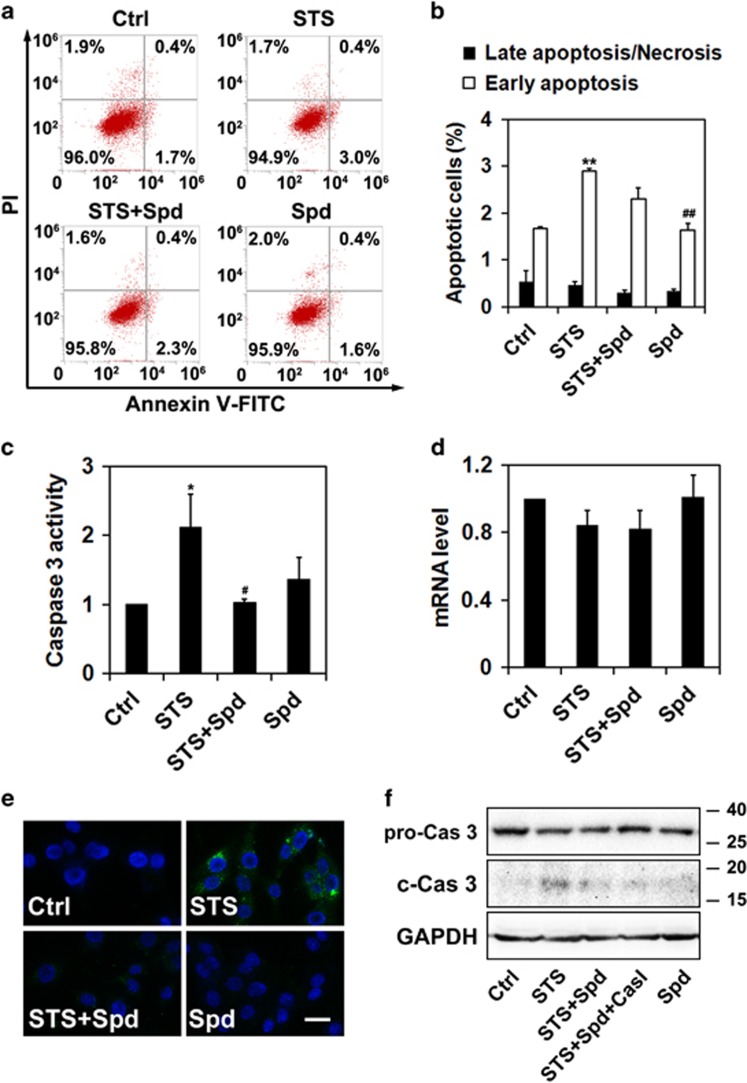
Spermidine inhibits caspase 3 activity. (**a**) Cell death was measured by Annexin V-FITC/PI double staining followed with flow cytometric analysis. Representative data were presented. (**b**) The percentages of early apoptotic and late apoptotic/necrotic cells were calculated from three independent experiments. (**c**) Caspase 3 activity in PC12 cells was measured by kit, and normalized to control. Data were calculated from six independent experiments. (**d**) mRNA level of caspase 3 was measured by real-time qPCR. The caspase 3 mRNA level was normalized by that of GAPDH. Data were calculated from three independent experiments. (**e**) Immunostaining of cleaved caspase 3 with an anti-cleaved caspase 3 antibody (green). Nuclei were counterstained with Hoechst 33342. Scale bar, 20 *μ*m. (**f**) Protein levels of pro-caspase 3 and cleaved caspase 3 were examined by western blotting. GAPDH was used as internal control. At least three independent experiments were conducted and representative immunoblots were presented. CasI, caspase inhibitor z-VAD-fmk. *P<0.05, **P<0.01 *versus* Ctrl; ^#^*P*<0.05, ^##^*P*<0.01 *versus* STS

To understand the mechanism of STS-induced cell damage, cell death was measured by Annexin V-fluorescein isothiocyanate (FITC)/propidium iodide (PI) staining followed with flow cytometric analysis. As revealed by Figures 2a and b, STS treatment resulted in increased percentage of early apoptotic cells (STS, 2.90±0.58% *versus* Ctrl, 1.67±0.03% *P*<0.01), which could be suppressed by the addition of spermidine (STS+Spd, 2.30±0.23%), albeit no statistically significant difference was detected when compared with STS alone (STS+Spd *versus* STS, *P*=0.065). Moreover, late apoptosis or necrosis did not occur in PC12 cells upon STS exposure. As the overall proportion of early/late apoptotic and necrotic cells was relatively small in STS-treated cells (<5%), apoptosis and necrosis may not be the major death mechanism at the early stage of STS incubation. Previous studies showed an enhanced caspase 3 activity in STS-treated PC12 cells.^15, 16^ Consistently, we also detected an increased caspase 3 activity in PC12 cells after STS exposure (*P*<0.05 *versus* Ctrl), which could be attenuated by co-administration with spermidine (*P*<0.05 *versus* STS) (Figure 2c). The mRNA level of caspase 3 was not significantly altered in the presence of STS and spermidine, alone or in combination, as evidenced by real-time quantitative PCR (qPCR) analysis (Figure 2d). Immunostaining studies, using an anti-cleaved (activated) caspase 3 antibody, showed strong immunoreactivity in cells exposed to STS, in comparison with the weak staining in other experimental groups (Figure 2e). Pretreatment with spermidine significantly declined the percentage of cells expressing cleaved caspase 3 (STS, 19.4±2.5% STS+Spd, 11.1±1.9% *P*<0.05). The endogenous protein levels of the pro-caspase 3 remained unchanged through all treatments (Figure 2f). However, increased caspase 3 cleavage was detected in STS-treated cells, and administration of spermidine or spermidine plus an pan-caspase inhibitor z-VAD-fmk blocked the caspase 3 activation.

### Spermidine promotes Beclin 1-dependent autophagic flux

**Figure 3:**
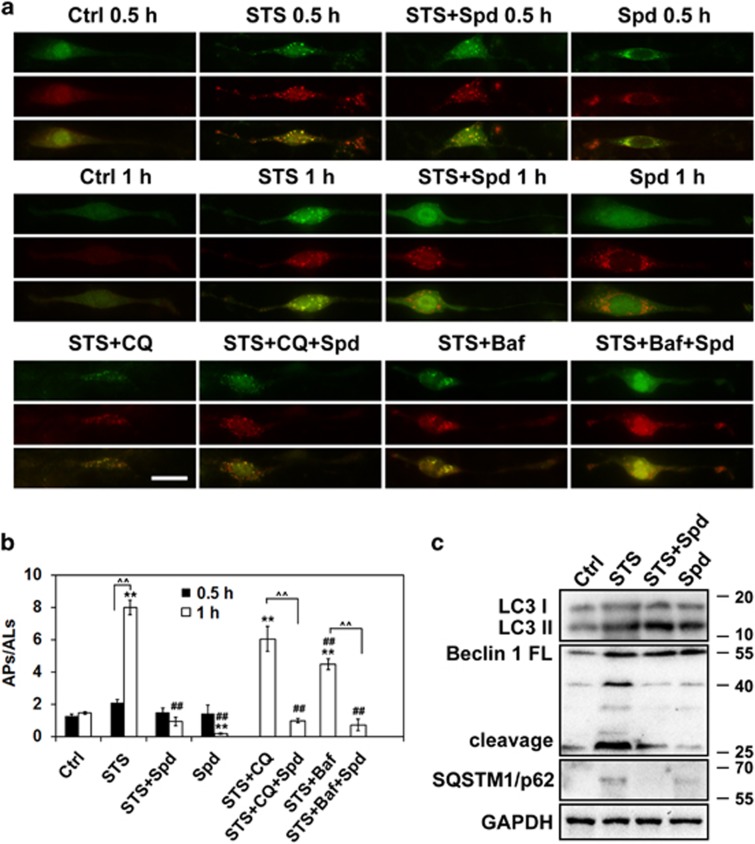
Spermidine promotes autophagic flux in STS-treated PC12 cells. (**a**) Cells were transfected with mRFP-GFP-LC3 plasmids, followed by 0.5 or 1 h of STS treatment in the presence or absence of autophagy blocker (CQ or BafA1). In STS+Spd, STS+CQ+Spd, STS+BafA1+Spd group, cells were pretreated with Spd for 1 h, followed by 1 h of combined STS, Spd with CQ or BafA1 incubation. Scale bar, 20 *μ*m. (**b**) The number of APs and ALs per cell was counted and the ratio of APs/ALs was presented. *P<0.05 *versus* Ctrl; ^##^*P*<0.01 *versus* STS. For each group, 15 cells were utilized for analysis and data were calculated from three independent experiments. (**c**) Western blotting analysis of LC3-I, LC3-II, Beclin 1 and SQSTM1/p62. GAPDH was used as internal control. At least three independent experiments were conducted and representative immunoblots were presented

**Figure 4:**
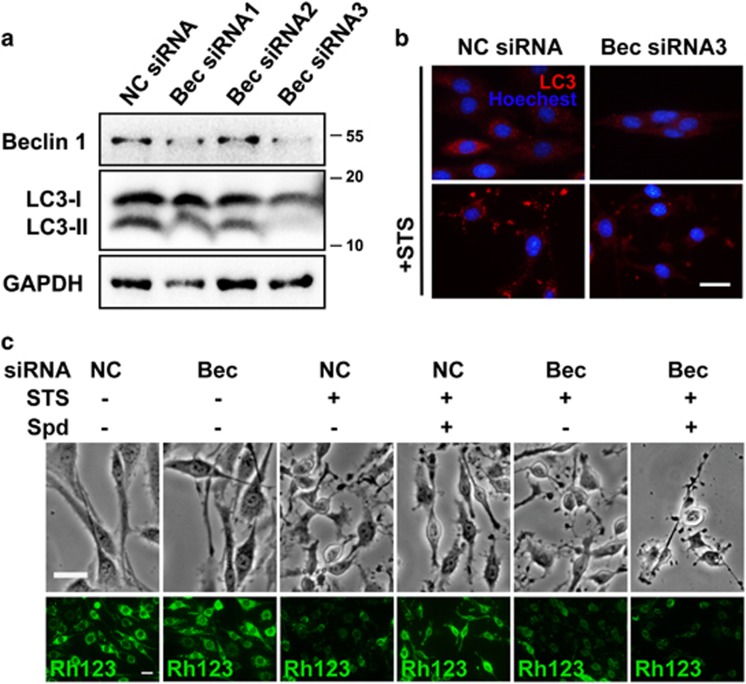
Influences of Beclin 1 silencing on STS-induced autophagy. (**a**) Western blotting analysis of Beclin 1 knockdown efficacy. Cells were transfected with NC siRNA, Beclin 1 siRNA1, siRNA2 or siRNA3. After 48 h, total protein was extracted for western blotting analysis. GAPDH was used as internal control. At least three independent experiments were conducted and representative immunoblots were presented. (**b**) Cells were transfected with NC siRNA or Beclin 1 siRNA3. After 48 h, some of the cells were exposed to STS. Immunostaining was carried out using an anti-LC3A/B antibody. Nuclei were counterstained with Hoechst 33342. (**c**) Morphology and mitochondrial membrane potential in Beclin 1-deficient cells. Cells were exposed to STS in the presence or absence of spermidine. Scale bar, 20 *μ*m

Accumulative studies indicate that the biological action of spermidine is closely associated with its capacity in inducing autophagy.^2, 5, 17, 18^ To elucidate whether or not the neuroprotective role of spermidine in STS-treated cells depends on autophagy modulation, we first tested the efficacy of other autophagy stimulators and inhibitors on cells in the presence of STS. Autophagy stimulator rapamycin (Rapa; 10 nM) also had a benifical effect against STS-induced neuritic shortening, whereas no protective effects were detected by using autophagy inhibitors, such as chloroquine (CQ; 10 *μ*M) and Bafilomycin A1 (BafA1; 40 nM) (Supplementary Figure S1). In addition, pretreatment with spermidine significantly alleviated STS-induced neuritic atrophy in the presence of CQ or BafA1. Second, autophagic flux was traced by transfecting cells with an mRFP-GFP-LC3 plasmid according to updated guidelines for the use and interpretations of assays for monitoring autophagy.^19^ In cells labeled with mRFP-GFP-LC3, yellow puncta (mRFP and GFP signals) represent autophagosomes (APs), whereas red puncta (mRFP only) represent autolysosomes (ALs). Incubation with STS for 0.5 h produced a pronounced increase of APs and ALs per cell (Figure 3a). With increased time of STS exposure (1 h), the autophagic puncta were accumulated in cells, and the ratio of APs/ALs was greatly elevated (Figures 3a and b), implying the dysfunctional autophagic flux in cells incubated with STS. Pretreatment with spermidine efficiently decreased the APs population in STS-treated cells. Combination of STS and an autophagy blocker (CQ or BafA1) also resulted in autophagic degradation failure, which could be attenuated by the pretreatment of spermidine. Western blotting analysis showed that STS treatment produced elevated expression levels of the lipid-bound LC3-II and autophagy substrate SQSTM1/p62, suggesting an insufficient autophagic clearance in STS-treated cells; whereas addition of spermidine ameliorated the dysfunction of autophagic degradation (Figure 3c). The LC3-II level remained high in the presence of STS and spermidine, which may be explained by the autophagic flux promotion capacity of spermidine. Interestingly, Beclin 1 cleavage occurred in cells exposed to STS, which could be reversed by the addition of spermidine. To illustrate whether or not STS-induced autophagy activation depends on Beclin 1, RNA interference was used to knockdown the intracellular Beclin 1 expression. Efficient silencing of Beclin 1 was detected in cells transfected with Beclin 1 siRNA3 (Figure 4a). Knocking down of Beclin 1 not only decreased baseline autophagy activation, but also strikingly suppressed STS-induced accumulation of LC3-positive puncta (Figures 4a and b), indicating the autophagic activity depends on Beclin 1. However, deficient in Beclin 1 expression could not suppress cell injury and mitochondrial membrane potential loss resulted from STS exposure (Figure 4c). Moreover, the protective effect of spermidine seems to be dependent on autophagy, as silencing of Beclin 1 abolished the neuroprotection of spermidine against STS. Collectively, these data suggest that spermidine prevents STS-induced cell death possibly through augmentation of Beclin 1-dependent autophagic flux.

### Caspase 3-mediated Beclin 1 cleavage possibly at the sites of 146 and 149 in STS-treated cells

**Figure 5:**
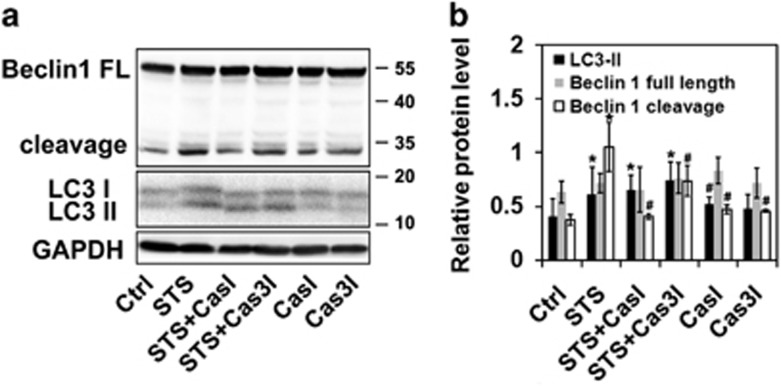
Caspase 3-mediated Beclin 1 cleavage in STS-treated PC12 cells. (**a**) Cells were treated with STS, STS+CasI, STS+Cas3I, CasI or Cas3I. The protein expression of Beclin 1, LC3 and GAPDH was evaluated by western blotting. (**b**) Relative protein expression (interested protein/GAPDH) was quantified. Data were calculated from five independent experiments. *P<0.05 *versus* Ctrl; ^#^*P*<0.01 *versus* STS

**Figure 6:**
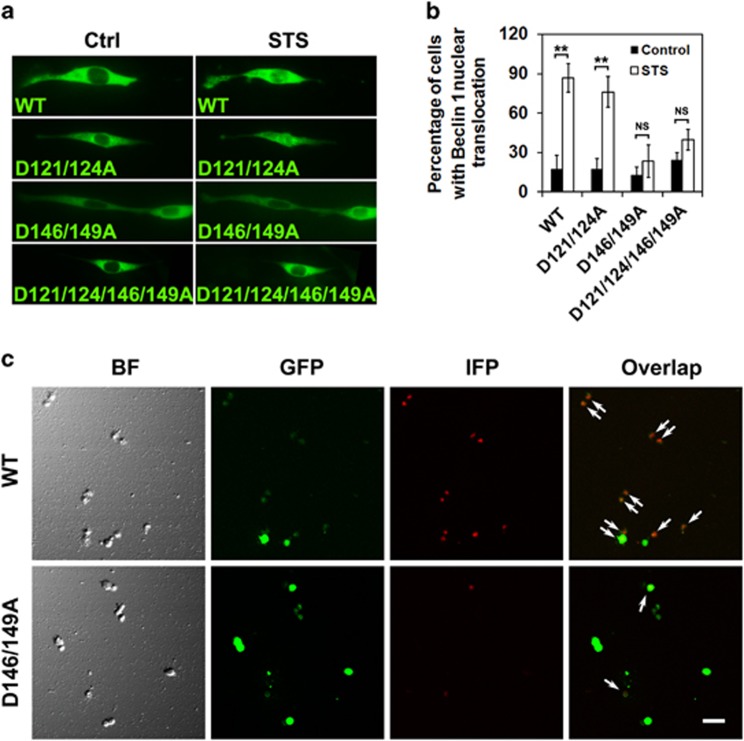
Mutation at the site of 146 and 149 prevented the nuclear translocation of Beclin 1 in cells exposed to STS. (**a**) Cells were transfected with WT Beclin 1 or mutated Beclin 1 (D121/124A, D146/149A and D121/124/146/149A) recombinant plasmids, followed with 1-h STS treatment. The fluorescence distribution was examined under fluorescence microscope equipped with a live cell station. Images of single cell before or after STS treatment were presented. (**b**) The average percentage of cells with Beclin 1 nuclear translocation was calculated. For each group, 10 images were used for analysis and data were calculated from three independent experiments. (**c**) iCasper confirms that D146/149A mutation is resistant to caspase cleavage. HEK293T cells were transfected with PLVX-iCasper (146/149 WT)-Puro (upper) or PLVX-iCasper (D146/149A mutant)-Puro (lower). After 1 h of STS treatment, reduced number of cells expressing infrared fluorescence of iCasper was detected in D146/149A mutant group. Cells with infrared fluorescence signals were indicated by arrows. BF, bright field; IFP, infrared fluorescent protein; GFP, green fluorescent protein. Scale bar, 20 *μ*m

To clarify the upstream executor of Beclin 1 cleavage, pharmacological caspase inhibitors were applied. Compared with STS group, pretreatment with the pan-caspase inhibitor z-VAD-fmk (CasI, 20 *μ*M) or a caspase 3-specific inhibitor Ac-DEVD-CHO (Cas3I, 20 *μ*M) reduced the levels of cleaved Beclin 1 in STS-incubated cells, leaving the LC3-II level unchanged (Figures 5a and b), indicating the caspase 3-mediated Beclin 1 cleavage occurs in response to STS. The caspase cleavage sites in Beclin 1 are highly conserved in diverse species (Supplementary Figure S2). Here, Beclin 1 wild type (WT), or mutations (aspartate [D]→alanine[A]) at 121/124, 146/149, 121/124/146/149, were inserted into pEGFP-C3 plasmids, in which the N-terminal Beclin 1 was tagged by EGFP (Supplementary Figure S3a). To investigate the potential effects of Beclin 1 mutation against caspase cleavage, cells were transfected with the constructed EGFP-Beclin 1-WT, EGFP-Beclin 1-D121/124A, EGFP-Beclin 1-D146/149A and EGFP-Beclin 1-D121/124/146/149A plasmids, and the distribution of fluorescent protein was monitored using a live cell station. In cells overexpressing WT Beclin 1, the nuclear translocation of Beclin 1 was detected after STS treatment (Figures 6a and b). D146/149A and D121/124/146/149A mutations, but not D121/124A, prevented Beclin 1 re-distribution, as well as caspase 3 activation in cells exposed to STS (Figures 6a and b,Supplementary Figure S4). The caspase-mediated cleavage at 146/149 sites of Beclin 1 was further confirmed using an infrared fluorescent executioner-caspase reporter (iCasper) (Supplementary Figures S3b-d). The iCasper was reported to become infrared fluorescent when cells underwent apoptotic cell death, and the number of cells expressing infrared fluorescent increased with time following STS incubation^20^ (see also Supplementary Figures S5). We redesigned the iCasper by incorporating 146–149 amino acids of WT (DQLD) or mutant (AQLA) Beclin 1. When the cleavage sequence is recognized and cleaved, infrared fluorescent would be detected. As expected, mutant 146/149 greatly decreased the number of cells expressing infrared fluorescent (Figure 6c), indicating the Beclin 1 mutation at the site of 146 and 149 is resistant to caspase cleavage.

### Spermidine prevents the nuclear translocation of Beclin 1 after cleavage

**Figure 7:**
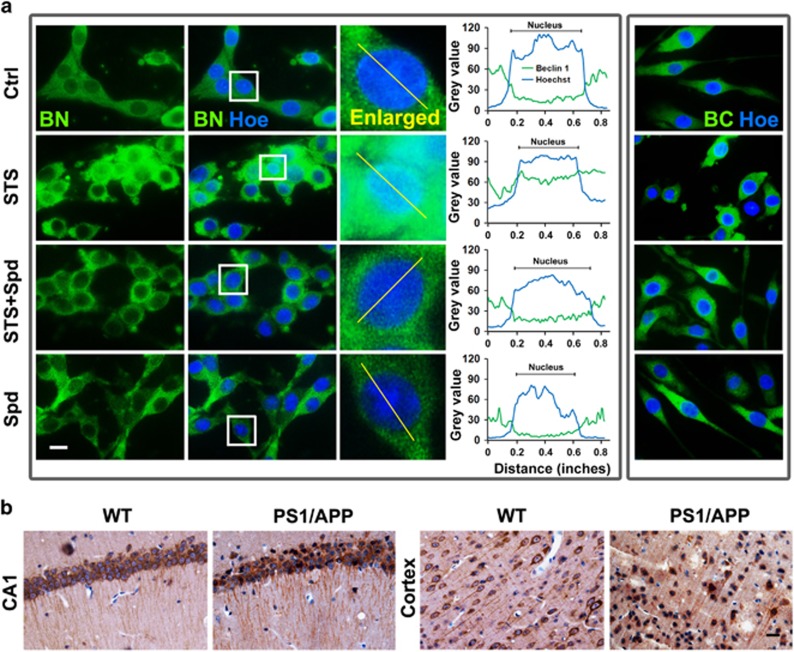
Nuclear translocation of Beclin 1 in damaged neurons. (**a**) Cells were treated with STS, STS+Spd or Spd. After incubation, cells were stained with anti-Beclin 1 N terminal (BN) or C-terminal (BC) antibody. The nuclei were counterstained with Hoechst 33342. Scale bar, 10 *μ*m. Curves indicated the colocalization between Beclin 1 (green) and Hoechst (blue) and correlated to the lines drawn in the enlarged images. The *x* axis represented the distance (inches) along the line and the *y* axis indicated the pixel intensity. (**b**) Hippocampal CA1 and cortical areas were obtained from 12-month-old PS1/APP (AD) mice (*n*=6) and age-matched WT control (*n*=6). Brain slides were immunostained with anti-Beclin 1 N terminal antibody and the nuclei were counterstained with hematoxylin. Scale bar, 20 *μ*m

Using an antibody specific against the N-terminal Beclin 1, we observed that Beclin 1 was mainly expressed in cytoplasm of healthy control cells. Upon STS stimuli, partial nuclear translocation of Beclin 1 N-terminus was detected in a large proportion of PC12 cells (Figure 7a). However, pretreatment with spermidine abolished the intracellular re-distribution of Beclin 1, possibly resulted from the blockage of Beclin 1 cleavage. Conversely, immunocytochemical studies using an antibody against the C-terminal Beclin 1 showed an apparently different expression pattern of C-terminus in cells exposed to STS, as C-terminal Beclin 1 was restricted in cytoplasm of most STS-treated cells. Note that inhibition of caspase 3 also blocked the nuclear translocation of Beclin 1 in cells treated with STS (Supplementary Figures S6). Transfection of GFP-labeled Beclin 1 full-length (GFP-BF), C-terminal (GFP-BC) or N-terminal fragment (GFP-BN) plasmid further confirmed the different distribution pattern of N- and C-terminal Beclin 1 in cells (Supplementary Figures S7). The nuclear translocation of Beclin 1 in damaged neurons appears to be a universal event among diversity of experimental paradigms. For instance, in hippocampal CA1 and cortex regions of PS1/APP mice, a rodent model of Alzheimer's disease (AD), the nuclear concentration of Beclin 1 was detected in a large proportion of neurons, compared with the cytosolic expression of Beclin 1 in the neurons of age-matched control brain (Figure 7b).

### Spermidine ameliorates I/R-induced neuronal injury *in vivo* possibly by inhibiting Beclin 1 cleavage

**Figure 8:**
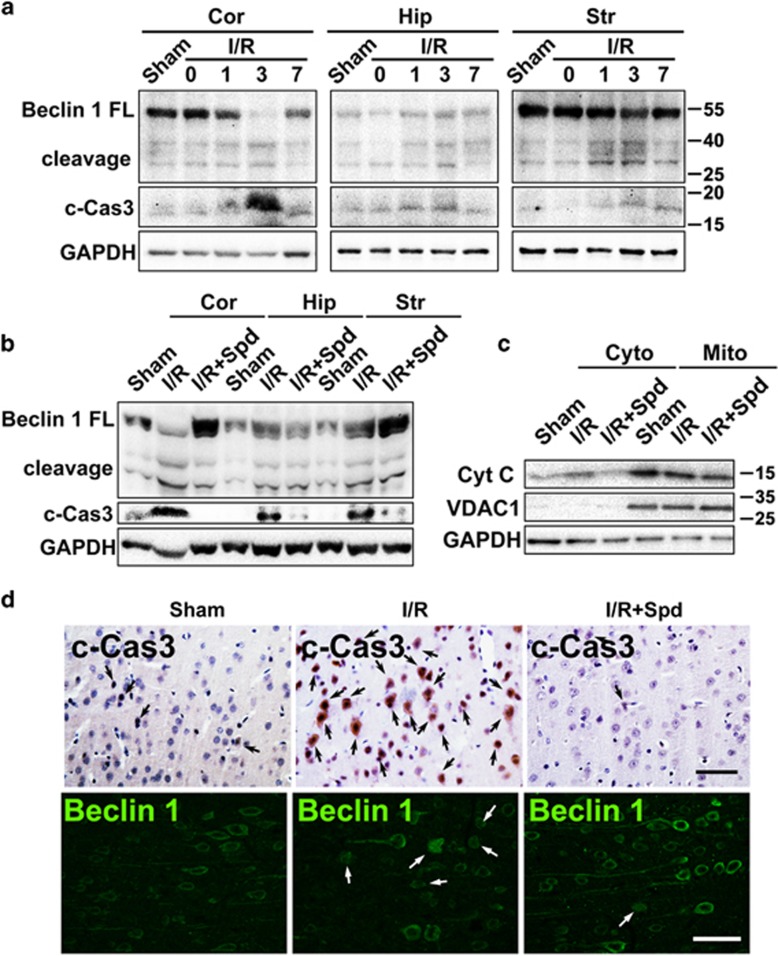
Spermidine prevented neuronal injury induced by I/R. (**a**) Animals received I/R injury, and the brain tissues were derived from cortex (Cor), hippocampus (Hip) or striatum (Str) at 0, 1, 3, 7 days following I/R. Brain samples were removed from Sham-operated animals at 3 days after surgery. The protein expression levels of Beclin 1, cleaved caspase 3 and GAPDH were examined by western blotting. (**b**) Three days following surgery, protein samples were extracted from animal brains in Sham operation, I/R or I/R+Spermidine (Spd) groups. (**c**) The cytochrome C concentration in cytosol or mitochondrial extracts was determined. VDAC1 and GAPDH were used as mitochondrial marker and internal control, respectively. (**d**) Immunostaining of cleaved caspase 3 or N-terminal Beclin 1 in cortex regions. Scale bar, 50 *μ*m. For each group, at least six animals were included; for each animal, at least four brain slices were used for analysis. Black arrows indicate the c-Cas3-positive cells, and white arrows indicate cells with nuclear translocation of Beclin 1

In order to convince the spermidine-mediated neuroprotection *in vivo*, rat global I/R injury model was established and a group of the model animals received intraperitoneal administration of spermidine. Compared with Sham operation control, I/R injury induced significant neuronal cell death in hippocampal CA1 region, as well as in cortex of animal brain (Supplementary Figure S8). Administration of spermidine relieved neuronal damage in both hippocampus and cortex. In addition, enhanced Beclin 1 cleavage and caspase 3 activation was noted in hippocampus, cortex and striatum areas after 3 days of I/R injury (Figure 8a). Spermidine greatly suppressed caspase 3 activation, inhibited Beclin 1 cleavage and the release of cytochrome C from mitochondria induced by I/R (Figures 8b and d). I/R injury triggered nuclear translocation of Beclin 1 in cortex, which could be reversed by spermidine therapy (Figure 8d). Interestingly, exogenous application of spermidine was capable in preventing the neuritic atrophy in rodent cerebrum following I/R injury. These data were in accordance with *in vitro* studies and collectively suggest that the neuroprotective function of spermidine may be yielded through inhibiting caspase 3-mediated Beclin 1 cleavage.

## Discussion

In this study, we investigated the involvement of caspase 3-mediated Beclin 1 cleavage during neuronal cell injury using cultured neuronal cells and rodents brain samples. Our results showed that Beclin 1 cleavage followed by partial nuclear translocation of N-terminal fragments occurred universally in damaged neuronal cells. Notably, administration of autophagy enhancer spermidine attenuated neuronal cell damage and caspase 3-induced Beclin 1 cleavage in cultured neuronal cells exposed to STS as well as in rat hippocampal and cortical neurons following I/R injury.

### Spermidine exerts neuroprotection in diverse experimental paradigms

The endogenous levels of polyamines, especially spermidine, decline continuously with age.^21^ Supplementation with spermidine ameliorates the age-induced memory impairment in fruit flies by triggering autophagy.^5^ In addition to anti-ageing activity, the neuroprotection of spermidine has also been reported in mitigating *α*-synuclein neurotoxicity, a pathological feature of Parkinson's disease, in model organisms including fruit flies and nematodes,^22^ and in rescuing the motor dysfunction of frontotemporal lobar dementia mice.^23^ Here, we provided direct evidence that spermidine protected against STS-induced acute neuronal cell damage in cultured PC12 cells and primary neurons. This notion was also confirmed by *in vivo* studies, which showed exogenous administration of spermidine reduced neuronal cell death in hippocampal CA1 and cortex of rat following I/R injury. Interestingly, application of spermidine prevented the neuritic atrophy in rodent cerebrum following I/R injury. Although the blood–brain barrier transport of spermidine is reported to be quite limited under physiological condition,^24^ Gilad *et al.*^25^ indicated that intravenous injection of spermidine caused an accumulation of this polyamine throughout the forebrain parenchyma after ischemia. Such phenomenon may be owned to the ability of spermidine in increasing the blood–brain barrier permeability under pathological states.^26^ Therefore, together with previous evidence, our findings suggest the promising therapeutic role of the natural polyamine for neurological disorders. One limitation of this study is the absence of spermidine treatment group on Sham-operated control rats, and therefore we could not speculate the potential effects of spermidine on normal animals.

### Spermidine restores autophagic flux

Emerging lines of evidence highlight the autophagy enhancing capacity of spermidine.^2, 4, 18, 22, 23, 27^ Spermidine triggers autophagy by suppressing the acetyltransferase activity of EP300,^28^ and inducing the acetylation or deacetylation of autophagy-related genes (Atgs).^29^ Nevertheless, the molecular mechanism by which spermidine induces neuronal autophagy remains largely unclear. To our knowledge, we here for the first time demonstrated that spermidine prevented caspase 3-mediated Beclin 1 cleavage and favored neuronal cell survival *in vitro* and *in vivo*. Treatment with STS led to an impaired autophagic clearance in PC12 cells, consisting with previous report, which shows AP accumulation in the early stage (within 12 h) after STS exposure in HeLa cells.^9^ The administration of spermidine enhanced Beclin 1-dependent autophagic flux and promoted the degradation of autophagy substrate SQSTM1/p62 in cells exposed to STS. Silencing of Beclin 1 abolished the neuroprotective action of spermidine against STS incubation. These results were in contrast with previous report, which shows a functional role of Beclin 1-independent autophagy in sensitizing cortical neurons to STS-induced apoptosis.^30^ The crosstalk between autophagy and apoptosis is therefore complex in governing the cell fate.^31^ Human Beclin 1 protein consists of three major domains, namely Bcl-2 homology domain 3 (BH3 domain), coiled-coil domain (CCD) and evolutionarily conserved domain (ECD), enabling its interaction with multiple proteins.^32^ AP formation depends on the normal binding of class III phosphoinositide 3-kinase (PI3K, Vps34) to the ECD of Beclin 1.^33^ However, N- or C-terminal fragment of Beclin 1, produced by cleavage, loses their ability to interact with PI3K/Vps34, which is required for autophagy induction.^11^ These data raise the possibility that spemidine facilitates functional autophagic clearance in injured cells by preserving full-length Beclin 1. However, we could not exclude the possibility that spermidine excerts its neuroprotective function via preserving the cleavage of other caspase 3 substrates apart from Beclin 1, including autophagy proteins, such as Atg7.^10^

### Spermidine inhibits caspase 3-mediated Beclin 1 cleavage

The effects of polyamines on caspase activation and cell apoptosis remain largely elusive. It is reported that spermine induces caspase 3 activation and triggers cell death through evoking cytochrome C release from mitochondria.^34^ In our preliminary study, prolonged incubation or increased dose of spermidine also resulted in caspase 3 activation and subsequent cell death (data not shown). Here, caspase 3 activation, an early event of cell apoptosis, occurred in PC12 cells at the early stage of STS incubation, whereas no abundant pycnotic nuclei were detected. In accordance with this result, it has been demonstrated that STS incubation led to early autophagy induction followed by apoptosis in HeLa cells.^9^ In addition, we found that pretreatment with 1 mM spermidine for 1 h efficiently suppressed caspase 3 activation induced by 1-h of STS exposure.

Cell-free *in vitro* studies reveal that Beclin 1 can be cleaved by several cell death proteases, such as caspase 3, caspase 6, caspase 8 and calpain 1.^10, 13, 14^ In our preliminary study, we examined the potential activation of caspase 3 and caspase 6 in PC12 cells exposed to STS, and evident caspase 3 cleavage, but not caspase 6 cleavage was detected (data not shown). Moreover, given that administration of pan-caspase inhibitor or caspase 3-specific inhibitor efficiently suppressed Beclin 1 cleavage, we speculate the caspase 3-induced Beclin 1 cleavage may have a pivotal role in damaged PC12 cells. These findings were in agreement with previous results obtained from mouse hematopoietic Ba/F3 cells^12^ and in human cervical carcinoma HeLa cells.^9^ However, we could not exclude the possibility that other cell death proteases are also involved in this process. Although several cleavage sites of Beclin 1 have been identified, including D^121^LFD^124^, TD^131^VD^133^ and D^146^QLD^149^,^10^ in our current PC12 injury model, it seems that D^146^QLD^149^, instead of D^121^LFD^124^, may be the cleavage site for caspase 3, as site mutation of D^146^QLD^149^ abolished STS-induced caspase 3 activation and Beclin 1 relocalization. Characterization of the crystal structure of the Beclin 1 sequence comprising D^146^QLD^149^ may offer insights into the preference of caspase cleavage in this site.

### Nuclear translocation of Beclin 1N terminal fragment during neuronal injury

Beclin 1 is mainly expressed in the cytoplasm of healthy neuronal cells. In cultured cells, as well as rodent brain tissues, a partial nuclear localization of Beclin 1 was coincidently detected in damaged neuronal cells. One possible explanation is the cleavage occurs at the site before 180aa, as Beclin 1 contains a leucine-rich nuclear export signal at amino acids 180–189.^35^ However, no classical nuclear localization sequence (NLS) is found in Beclin 1. Therefore, the N-terminal Beclin 1 fragment may be imported into the cell nucleus via certain non-classical NLS. Indeed, a potential NLS of Beclin 1 may be E^110^NLSRRLKV^118^, which highly enriched in basic amino acids (K, R), although still needs to be further clarified by site mutation studies. The function of N-terminal Beclin 1 fragment after nuclear translocation is unclear. One possibility may be associated with DNA regulation, as Beclin 1 has been implicated to regulate DNA damage and centrosome stability in colorectal cancer cells.^36^ Regarding the C-terminal fragment, it has been demonstrated that the C-terminal Beclin 1 sensitized the Ba/F3 cells to apoptosis by driving the release of cytochrome C from mitochondria.^12^ It is reasonable, therefore, to assume that after cleavage, C-terminal Beclin 1 gains novel function of augmenting cell apoptosis via inducing the release of pro-apoptotic factor from dysfunctional mitochondria.

Taken collectively, our study elucidates that spermidine prevents neuronal injury by inhibiting caspase 3-mediated Beclin 1 cleavage and subsequently restoring functional autophagic flux. These findings shed new light on the molecular mechanism of spermidine-modulated neuroprotection and provide a reference for the spermidine-based drug development.

## Materials and methods

### Reagents

Dulbecco's modified Eagle's medium (DMEM) was obtained from Biological Industries Israel Beit-Haemek Ltd, Kibbutz Beit-Haemek, Israel. Heat-inactivated horse serum (hiHS) was purchased from Thermo Fisher Scientific, Auckland, New Zealand. Minimal essential medium (MEM), opti-MEM medium, Neurobasal medium, B27 supplements, Rhodamine 123, Lipofectamine RNAiMAX reagent, TRIzol reagent, RevertAid reverse transcriptase, dNTP and RiboLock RNase inhibitor were purchased from Thermo Fisher Scientific, Grand Island, NY, USA. Fetal calf serum (FCS) was obtained from Hangzhou Tianhang Biological Technology Co., Ltd, Hangzhou, China. STS, caspase 3 activity assay kit, Bradford protein quantitative kit, and bicinchoninic acid (BCA) protein assay kit were bought from Beyotime Institute of Biotechnology, Haimen, Jiangsu, China. pEGFP-C3 was obtained from Clontech (Mountain View, CA, USA). Pan-caspase inhibitor z-VAD-fmk (CasI) and caspase 3-specific inhibitor Ac-DEVD-CHO (Cas3I) were purchased from EMD Biosciences, Inc., San Diego, CA, USA. Poly-d-lysine, cytosine *β*-D-arabinofuranoside (AraC), spermidine and CQ were bought from Sigma-Aldrich (Shanghai) Trading Co., Ltd, Shanghai, China. Rapa and BafA1 were obtained from Alexis Biochemicals, San Diego, CA, USA.

### Cell culture

Differentiated rat pheochromocytoma PC12 cells and human embryonic kidney 293 cells that contain the SV40 large T-antigen (HEK293T) were bought from the Institute of Cell Biology, Chinese Academy of Sciences (Shanghai, China). PC12 cells were cultured in DMEM containing 10% hiHS and 5% FCS and HEK293T cells were maintained in DMEM containing 10% FCS. Cortical neurons were prepared from newborn rat pups as previously described^37^ with minor modifications. In brief, cortices were removed and dissociated in trypsin solution. Dissociated cells were suspended in MEM containing 5% hiHS and 5% FCS, and plated onto coverglass bottom dish pre-coated with poly-d-lysine. On the next day, culture medium was replaced with neurobasal medium containing B27 supplements and AraC. Cells were incubated at 37 °C in a 5% CO_2_ atmosphere and 95% humidity.

### Drug treatment

To induce neuronal injury, cells were incubated with 1 *μ*M STS in culture medium for 1 h. For pharmacological interference, cells were pretreated with spermidine (1 mM), CasI (20 *μ*M), spermidine (1 mM)+CasI (20 *μ*M), Cas3I (20 *μ*M), spermidine (1 mM)+Cas3I (20 *μ*M), Rapa (10 nM), Rapa (10 nM)+CasI (20 *μ*M), CQ (10 *μ*M), CQ (10 *μ*M)+spermidine (1 mM), CQ (10 *μ*M)+CasI (20 *μ*M), BafA1 (40 nM), BafA1 (40 nM)+spermidine (1 mM), or BafA1 (40 nM)+CasI (20 *μ*M) for 1 h, followed by STS (1 *μ*M) co-administration for another 1 h. Some cells were exposed to stimulators or inhibitors in the absence of STS for 2 h. Cells without drug incubation were used as negative control. Experiments were performed in triplicate.

### Determination of the neuritic length and nuclear diameter

After treatment, cells were fixed in 4% paraformaldehyde (PFA) and were examined under phase contrast microscope (Eclipse Ts 100, Nikon, Tokyo, Japan) at × 200 magnification. To examine nuclear diameter, cells were stained with Hoechst 33342 dye. Ten non-overlapped micrographs were captured from each sample. The neuritic length and nuclear diameter were measured by ImageJ 1.47e software (Wayne Rasband, National Institutes of Health, Bethesda, MD, USA). Six non-overlapped neurites/nuclei were randomly selected from each micrograph, the average neuritic length and nuclear diameter (*μ*m) were calculated from 60 neurites and 60 nuclei, respectively.

### Assessment of mitochondrial membrane potential

Mitochondrial membrane potential was evaluated by Rhodamine 123 staining. Rhodamine 123 (100 *μ*M) was diluted in pre-warmed DMEM culture medium to a working concentration of 0.5 *μ*M. The culture medium was removed, and cells were incubated with the staining solution for 30 min in dark at 37 °C. After three times washing with DMEM, the fluorescence intensity was examined under fluorescent microscope at × 400 magnification. The average integrated optical density (IOD) per area was determined using ImageJ 1.47e software.

### Cell death analysis

Cell death was measured using Annexin V-FITC/PI apoptosis detection kit according to the manufacturer's instructions (Multisciences, Hangzhou, China). A total of 1 × 10^5^ cells were analyzed with Attune NxT flow cytometer (AFC2, Thermo Fisher Scientific, Eugene, OR, USA). The average percentages of early apoptotic cells (Annexin V^+^/PI^−^) and late apoptotic/necrotic cells (Annexin V^+^/PI^+^) were calculated from three independent experiments.

### Measurement of caspase 3 activity

Caspase 3 activity assay kit was used for the measurement of caspase 3 enzyme activity. Briefly, 50 *μ*l cell lysis buffer was mixed with 10 *μ*l Ac-DEVD-pNA (2 mM) and 40 *μ*l buffer and loaded into a 96-well plate. After incubating at 37 °C for 4 h, the absorbance was measured at 405 nm by a microplate reader (Spectrophotometer 1510, Thermo Fisher Scientific, Vantaa, Finland). The caspase 3 activity in each sample solution was calculated by standard curve method. The final results are normalized by the quantity of total protein using Bradford protein quantitative kit.

### Real-time qPCR

Real-time qPCR was performed to examine the mRNA level of caspase 3. Total RNA was extracted from cultured PC12 cells using TRIzol reagent. The integrity of RNA was evaluated by agarose gel electrophoresis. For each sample, 10–1000 ng of total RNA was reverse transcribed with the RevertAid reverse transcriptase, dNTP and RiboLock RNase inhibitor (Fermentas, Thermo Fisher Scientific, Waltham, MA, USA). Synthesized cDNA was then used for real-time qPCR amplification on ABI stepone plus PCR equipment (Applied Biosystems, Foster City, CA, USA). Primers including *caspase 3*, forward, 5′-TCTGACTGGAAAGCCGAAACT-3′ reverse, 5′-CCATGACCCGTCCCTTGA-3′ *gapdh*, forward, 5′-CGCTAACATCAAATGGGGTG-3′ reverse, 5′-TTGCTGACAATCTTGAGGGAG-3′, were synthesized by Invitrogen Trading (Shanghai) Co., Ltd, Shanghai, China. The PCR reaction consisted of a denaturation step at 95 °C for 3 min, followed by 40 cycles at 95 °C for 3 s and 56 °C for 30 s.

### Western blotting

Cells or tissue samples derived from cortex, hippocampus or striatum of the rat brains were collected and lysed on ice. After centrifuging, supernatants were collected and the protein concentration was determined using BCA protein assay kit. Equal amount of proteins were separated on 12% or 15% SDS-PAGE, transferred onto a PVDF membrane, blocked with 5% bovine serum albumin solution and probed with primary antibodies (Supplementary Table 1) overnight at 4 °C. After washing, membranes were incubated with mouse/rabbit horseradish peroxidase (HRP)-conjugated secondary antibodies at room temperature for 2 h, and then detected with an enhanced chemoluminescence detection kit (Pierce, Rockford, lL, USA). Images were acquired by ChemiDoc XRS+ Imaging System (Bio-Rad, Hercules, CA, USA) and visualized using Image Lab version 4.1 software (Bio-Rad).

### Plasmid reconstruction

pCDNA4TO-/Myc.his B vectors containing human full-length (WT) Beclin 1, double mutant (D121A/D124A, D146A/D149A; aspartate 'D'→alanine 'A' at indicated sites), and quadruple mutant (D121A/D124A/D146A/D149A) Beclin 1 were provided by Dr. Yushan Zhu from Nankai University, Tianjin, China. WT or mutant Beclin 1 sequences were digested by *Hind*III and *Bam*HI restriction enzymes, cloned into pEGFP-C3 vectors, and sequenced, respectively. Four recombinant plasmids (EGFP-Beclin 1-WT, EGFP-Beclin 1-D121/124A, EGFP-Beclin 1-D146/149A and EGFP-Beclin 1-D121/124/146/149A) were identified by DNA sequencing.

The iCasper sequence (N-splitGFP-GAF-Linker-PAS-C-splitGFP) together with T2A-HO1 sequence was cloned from pcDNA3.1-iCasper-T2A-HO1, provided by Xiaokun Shu, University of California, San Francisco (UCSF), San Francisco, CA, USA.^20^ To clarify the caspase cleavage site, the key amino-acid sequence (the 'Linker' between GAF and PAS domains) was changed to DQLD or AQLA using overlapping PCR. Then the redesigned iCasper (146/149 WT or D146/149A mutant) sequence was inserted into linearized PLVX-Puro vector (Clontech), creating a PLVX-iCasper (146/149 WT or D146/149A mutant)-Puro plasmid.

### Transfection

To evaluate the autophagic flux, cells were transfected with mRFP-GFP-LC3 plasmid provided by Tamotsu Yoshimori (Osaka University, Osaka, Japan). To understand the intracellular distribution of Beclin 1 full-length and fragments, cells were transfected with Beclin 1-EGFP, Beclin 1N-EGFP (1–149 aa), or Beclin 1-C-EGFP (150–450 aa) plasmids provided by David Rubinsztein (University of Cambridge, Cambridge, UK). To investigate the potential effects of Beclin 1 mutation against caspase cleavage, cells were transfected with EGFP-Beclin 1-WT, EGFP-Beclin 1-D121/124A, EGFP-Beclin 1-D146/149A, EGFP-Beclin 1-D121/124/146/149A, pcDNA3.1-iCasper-T2A-HO1, PLVX-iCasper (146/149 WT)-Puro or PLVX-iCasper (D146/149A mutant)-Puro plasmids. Transient transfection was carried out using TurboFect transfection reagent according to the manufacturer's instructions (Thermo Scientific, Waltham, MA, USA). Cells were examined by fluorescent microscope (IX-81, Olympus, Tokyo, Japan) equipped with live cell station, which kept optimal temperature, humidity, CO_2_ gas concentration during experiment, under × 600 magnification. In some experiments, the transfected cells were fixed and probed with certain primary antibody for the analysis of immunocytochemistry. In iCasper experiments, cells were examined by laser confocal microscope (FV1200, Olympus) under × 200 magnification. The wavelengths in 488 nm and 635 were used as excitation sources for GFP and iRFP, respectively.

### RNA interference

To knockdown the Beclin 1 expression, three siRNAs targeting Beclin 1 were synthesized by Oligobio Co., Ltd, Beijing, China. The sequences of siRNAs were as follows: siRNA1: sense, 5′-GGCACGAUCAAUAAUUUCAtt-3′, anti-sense, 5′-UGAAAUUAUUGAUCGUGCCtt-3′ siRNA2: sense, 5′-GAGGAGCCAUUUAUUGAAAtt-3′, anti-sense, 5′-UUUCAAUAAAUGGCUCCUCtt-3′ siRNA3: sense, 5′-CUCAGGAGAGGAGCCAUUUtt-3′, anti-sense, 5′-AAAUGGCUCCUCUCCUGAGtt-3′ negative control (NC): sense, 5′-UUCUCCGAACGUGUCACGUtt-3′, anti-sense, 5′-ACGUGACACGUUCGGAGAAtt-3′. siRNAs were transfected into PC12 cells using Lipofectamine RNAiMAX reagent. Forty-eight hours after transfection, the silencing efficacy was assessed by determination of the Beclin 1 protein expression.

### Immunocytochemistry

PFA-fixed cells were permeabilized, blocked and then incubated with primary antibody in a humidified chamber overnight at 4 °C. The primary antibodies used for immunocytochemistry were listed in Supplementary Table 1. After phosphate-buffered saline washing, cells were probed with mouse/rabbit Alexa Fluor 488/546-conjugated secondary antibodies for 1.5 h at room temperature. Nuclei were counterstained with Hoechst 33342. Samples were examined under fluorescent microscope (BX51, Olympus) at × 600 magnification. The average percentage of immunopositive cells over total number of cells was calculated. To investigate the nuclear translocation of Beclin 1, cells were immunostained with anti-Beclin 1 (NT) antibody, and the nuclei were counterstained with Hoechst. The nuclear translocation of Beclin 1 was measured by ImageJ 1.47e software. A straight line was made across the nucleus in micrograph, and the fluorescence intensity along the line was measured. Plot Profile and Excel were used to plot the results.

### Cerebral I/R model and drug administration

Male SD rats, weighing 200–240 g, were obtained from Animal Center of Hangzhou Normal University, Hangzhou, China. A total of 70 were assigned for three groups, Sham operation group (*n*=17), I/R group (*n*=37) and I/R+Spd group (*n*=16). Transient global cerebral ischemia was induced by common carotid artery occlusion combined with systemic hypotension in rats. In brief, animals were anesthetized with 10% chloral hydrate (350 mg/kg) intraperitoneally. A ventral midline neck incision was made and bilateral common carotid arteries were exposed by blunt dissection. After intraperitoneal administration of 0.1% sodium nitroprussiate (2 mg/kg), common carotid arteries were clamped by microaneurysm clips for 20 min. Then, the clips were carefully removed for blood reperfusion. In Sham operation group, bilateral common carotid arteries were exposed but not occluded. The incisions were sutured after surgery. Animals in I/R+Spd group were given 10 mg/kg Spd (0.1 ml/100 g) intraperitoneally 30 min before, at 24 and 48 h after cerebral ischemia. I/R rats and Sham-operated rats were injected with the same volume of saline. The animal experiments were approved by the Animal Ethics Committee of Hangzhou Normal University.

### PS1/APP and age-matched control mice

Twelve-month old APP/PS1 transgenic mice (*n*=6) and age-matched WT C57BL/6 mice (*n*=6) were purchased from National Resource Center of Model Mice (NRCMM) and Model Animal Research Center of Nanjing University (MARC), Nanjing, China. The mice brains were removed and fixed in 4% PFA until use.

### Immunohistochemistry and immunostaining

Tissue samples were fixed in 4% PFA, dehydrated in ethanol series, cleared in xylene, embedded in paraffin and sectioned into 5-*μ*m-thick slices. Some of the sections were stained with hematoxylin and eosin (HE) for histological examination. Others were probed with specific primary antibodies (Supplementary Table 1) at 4 °C overnight. For immunohistochemical analysis, samples were stained with HRP-conjugated anti-rabbit or anti-mouse IgG secondary antibody and examined using the Polink-1 HRP DAB detection kit according to the manufacturer's instructions (Zhongshan GoldenBridge Biotechnology, Co., Ltd, Beijing, China). Immunoreaction was analyzed under light microscope. For immunostaining, samples were stained with Alexa Fluor secondary antibodies. Immunoreaction was examined under fluorescent microscope at × 400 magnification. At least six animals from each group were included. For each animal, at least four brain slices were used for analysis.

### Examination of cytochrome C releasing

In order to determine the cytochrome C releasing from the mitochondria into cytosol, mitochondria/cytosol fractionation was performed using cell mitochondria isolation kit according to the manufacturer's instructions (Beyotime Institute of Biotechnology, Haimen, Jiangsu, China). Briefly, cells were collected and resuspended in mitochondria/cytosol fractionation solution containing phenylmethanesulfonyl fluoride (PMSF). Cell samples were homogenized in a glass tissue homogenizer. After centrifuging at 800 *g* for 10 min at 4 °C, the supernatant was collected into a clean pre-chilled tube and was centrifuged again at 11 000 *g* for 10 min at 4 °C. The supernatant was collected as cytosol extracts. The pellet was resuspended in mitochondrial extraction buffer containing PMSF. After vortex, the mixture was incubated for 30 min on ice followed by 12 000* g* centrifuging for another 30 min at 4 °C. The supernatant was saved as mitochondrial extracts.

### Statistical analysis

Data were calculated from at least three independent experiments and were presented as means±S.E.M. Comparison between two groups was carried out using Student's *t*-test. One-way analysis of variance (ANOVA) followed by Bonferroni's test was utilized for multiple comparisons. *P*<0.05 or *P*<0.01 was recognized as significantly different.
