# Supplementary material for: Induction of autophagy by spermidine is neuroprotective via inhibition of caspase 3-mediated Beclin 1 cleavage
Source: Cell Death Dis. 2017 Apr 6;8(4):e2738–. doi: 10.1038/cddis.2017.161 (PMC5477584; doi:10.1038/cddis.2017.161)
Supplement: Supplementary Information [file cddis2017161x1.docx]

**Figure S1** Effects of autophagy stimulator/inhibitor on STS-induced cell death. Cells were incubated with chloroquine (CQ), STS+CQ, STS+CQ+Spd, Bafilomycin A1 (BafA1), STS+BafA1, STS+BafA1+Spd, rapamycin (Rapa), or STS+Rapa. (**a**) Micrographs were captured under light microscope. Scale bar, 50 μm. (**b**) The mean neuritic length. For each group, 60 cells were ultilised for analysis and data were calculated from three independent experiments. ^**^*P*<0.01 *vs.* Ctrl; ^#^*P*<0.05, ^##^*P*<0.01 *vs.* STS; ^^*P*<0.01.


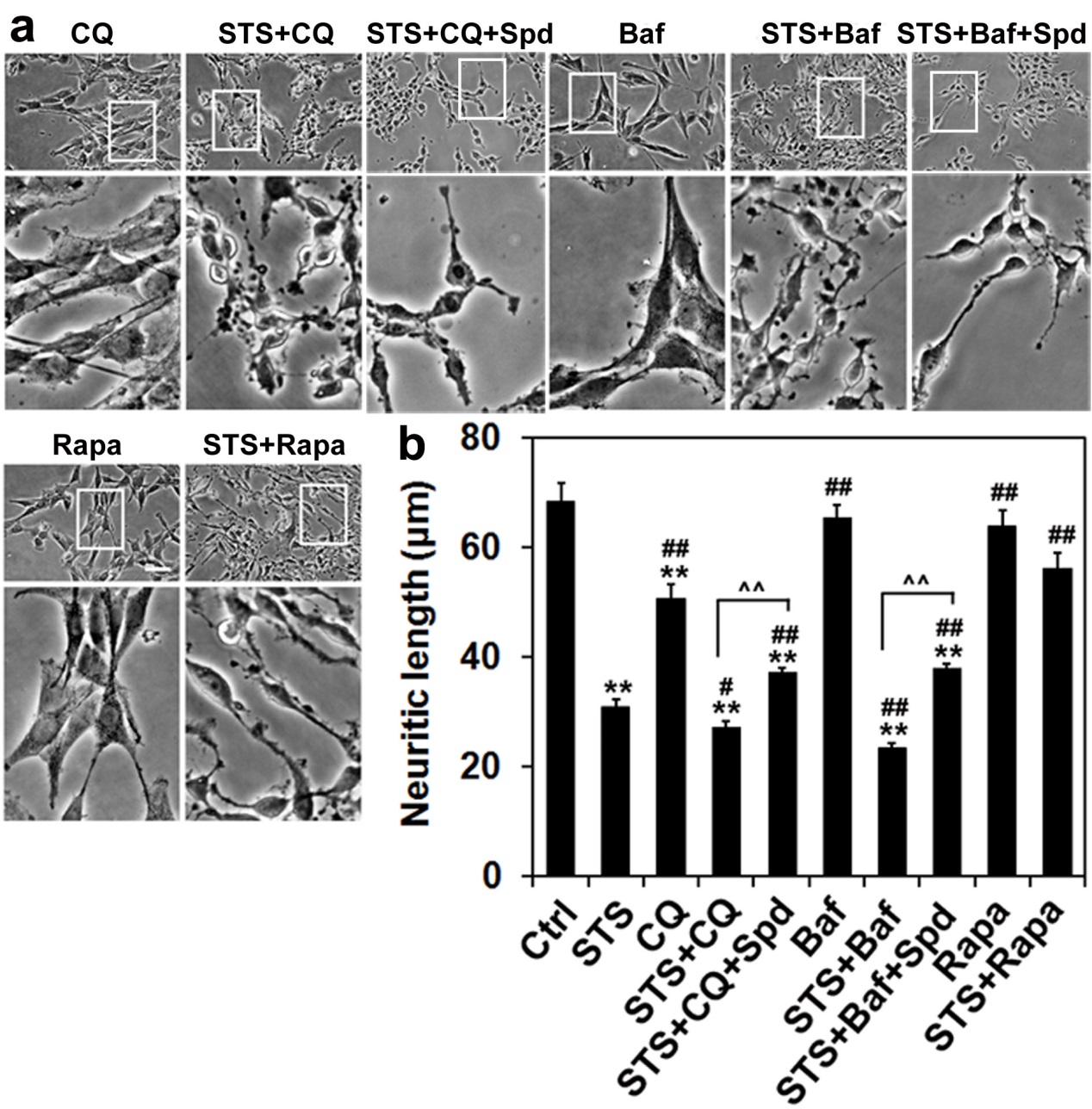


**Figure S2** A schematic representation and amino acid sequences of Beclin 1. For comparison, the sequences of Beclin 1 derived from different species were imported into Vector NTI Advance 11.5.1 (Invitrogen), and the alignment was generated using AlignX program.


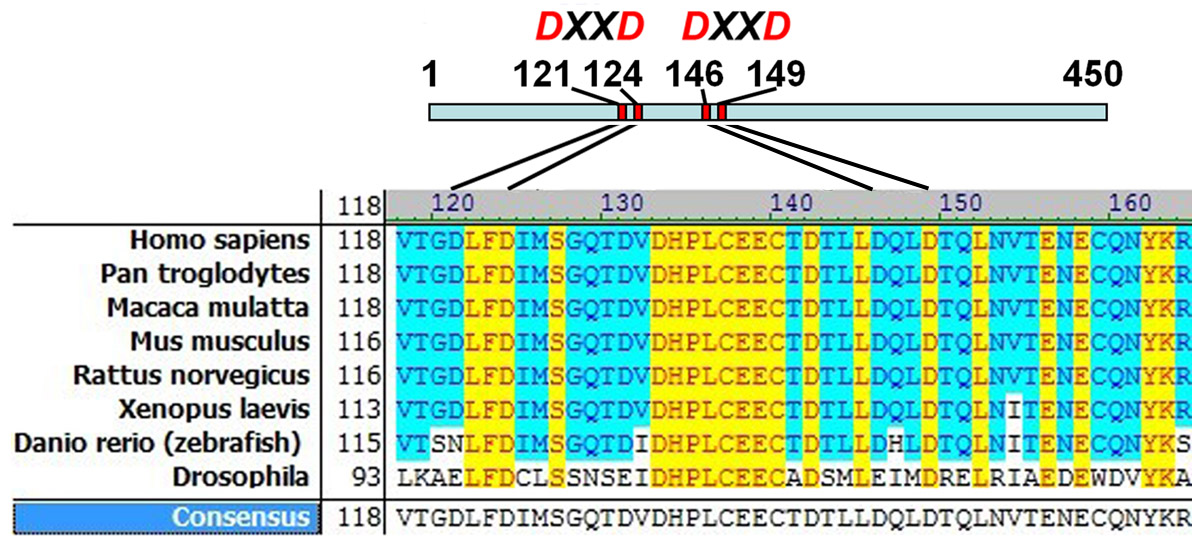


**Figure S3** Plasmid construction. (**a**) Beclin-1 and its mutants were cloned from pcDNA4/TO-Beclin-1-His/myc and inserted into pEGFP-C3 vector. (**b**) The iCasper sequence (N-splitGFP-GAF-Linker-PAS-C-splitGFP-T2A-HO1) was modified from pcDNA3.1-iCasper-T2A-HO1 and inserted into PLVX-Puro vector. (**c**) Before cleavage, the PAS and GAF domains were separated by the Linker. The iCasper shows green fluorescence only due to the self-assembling of splitGFP. (**d**) After cleavage, the PAS and GAF domains formed into integrated iRFP, and the iCasper gave both green and inferred fluorescence.


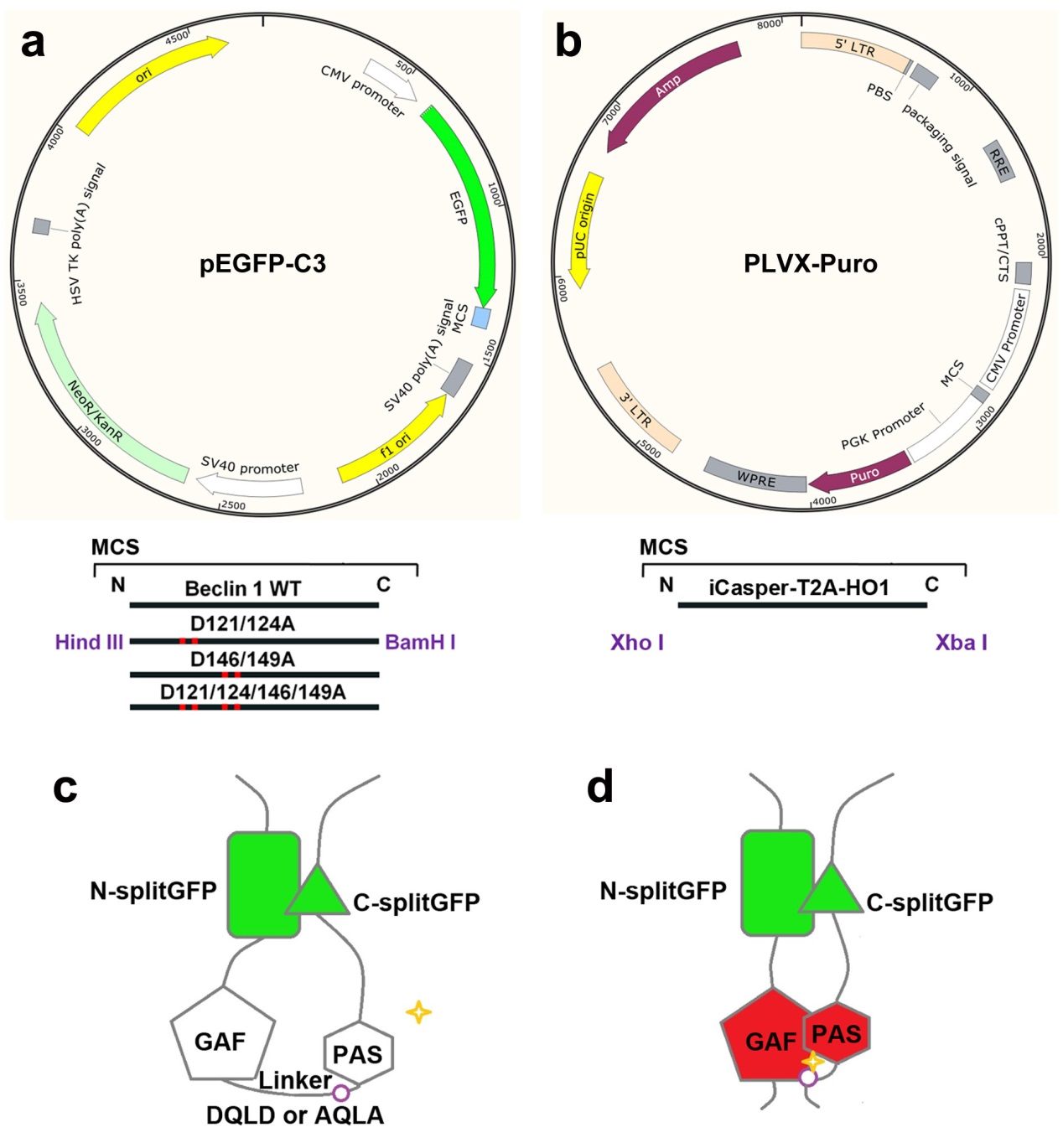


**Figure S4** Mutation at the site of 146 and 149 prevented STS-induced Caspase 3 activation. Cells transfected with different plasmids were exposed to STS. After incubation, cells were immunostained with anti-c-Cas3 antibody. Nuclei were counterstained with Hoechst. Scale bar, 10 μm.


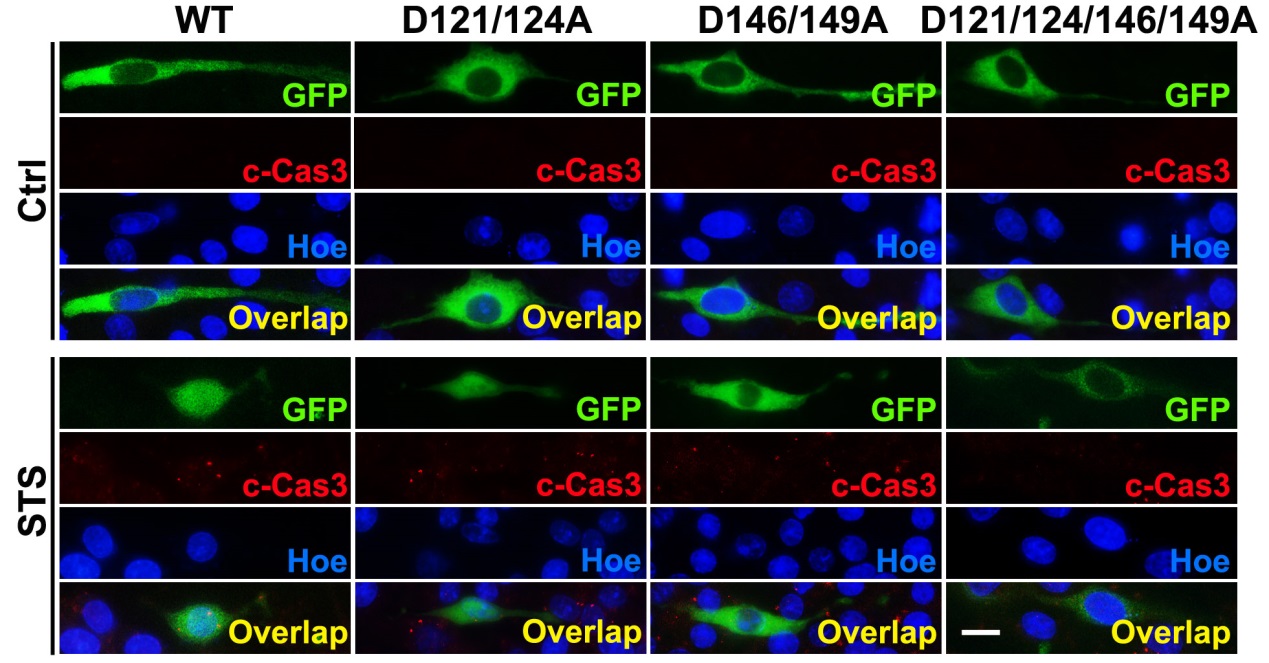


**Figure S5** HEK293T cells expressing iCasper. HEK293 cells were transfected with pcDNA3.1-iCasper-T2A-HO1. After the number of cells expressing infrared fluorescence of iCasper was increased with time following STS exposure. Cells with infrared fluorescence signals were indicated by arrows. BF, bright field; IFP, infrared fluorescent protein; GFP, green fluorescent protein. Scale bar, 20 μm.


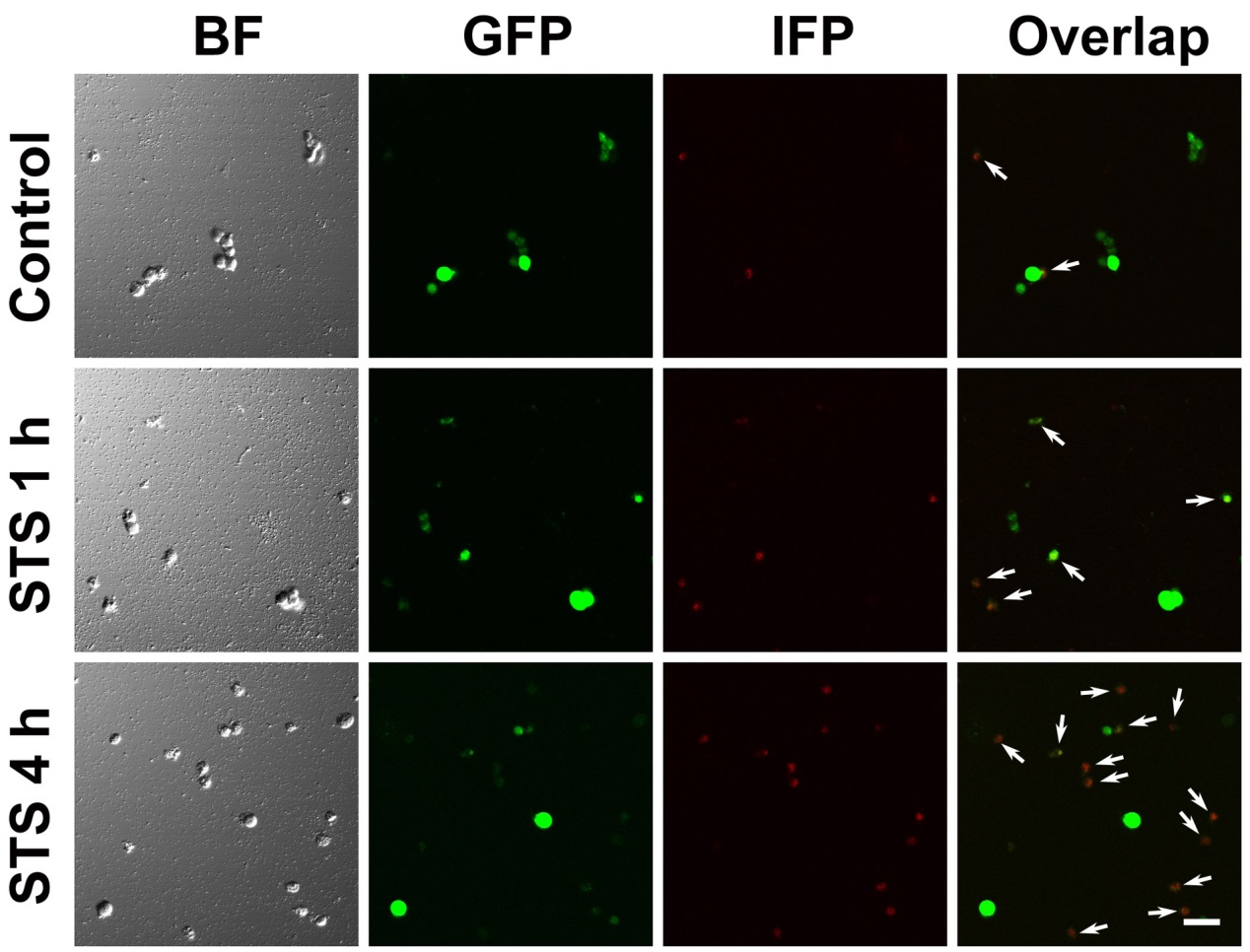


**Figure S6** Caspase inhibitor prevented nuclear translocation of Beclin 1. Cells were treated with STS, STS+CasI, or STS+Cas3I. After incubation, cells were stained with anti-Beclin 1 N-terminal (BN) or C-terminal (BC) antibody. The nuclei were counterstained with Hoechst 33342. Scale bar, 10 μm. Curves indicated the colocalization between Beclin 1 (green) and Hoechst (blue) and correlated to the lines drawn in the enlarged images. The x-axis represented the distance (inches) along the line and the y-axis indicated the pixel intensity.


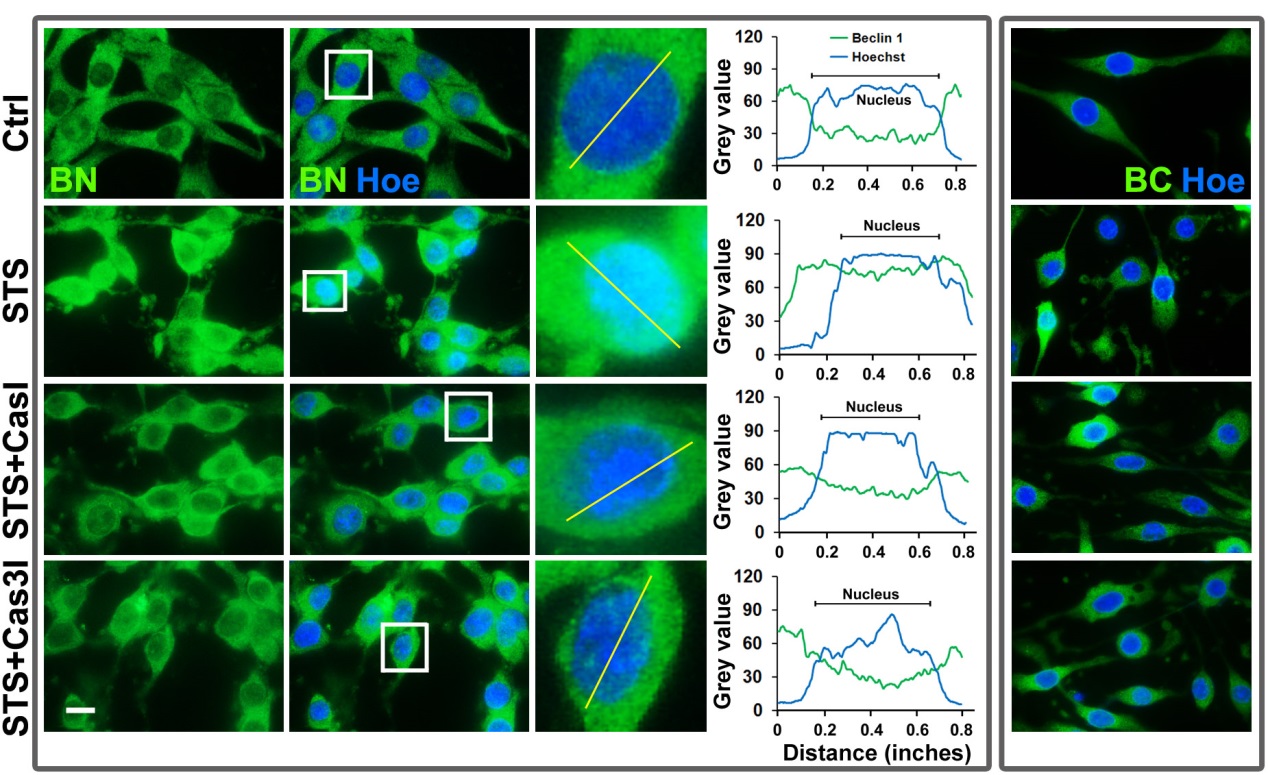


**Figure S7** Intracellular distribution of Beclin 1 fragments. PC12 cells were transfected with GFP-labeled Beclin 1 full length (GFP-BF), C- (GFP-BC), or N-terminal fragment (GFP-BN) plasmid. One day after transfection, cells were fixed and nuclei were stained with Hoechst 33342. Samples were examined under fluorescent microscope. Scale bar, 20 μm.


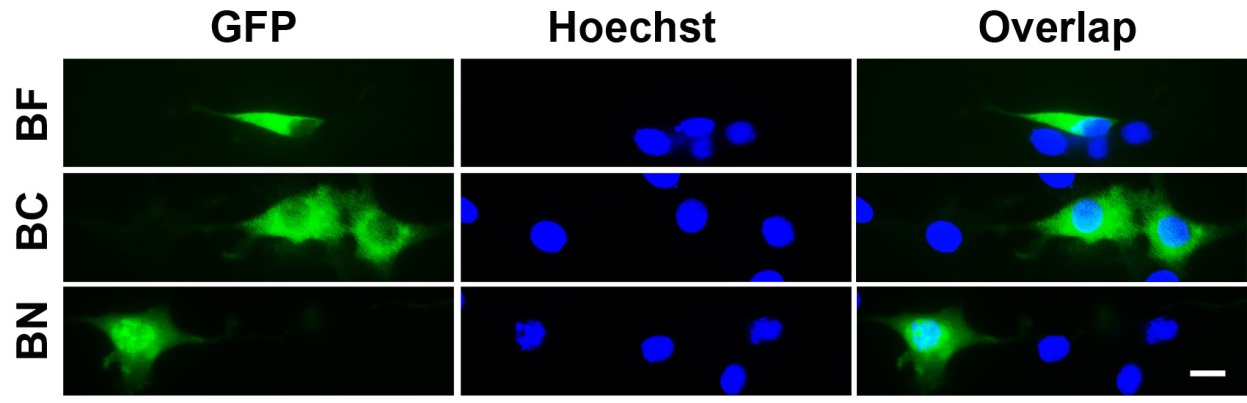


**Figure S8** Histological examination. Brain samples derived from animals in Sham operation, I/R or I/R+Spermidine (Spd) groups were stained with HE for histological examination. (**a**) Cell damage in hippocampal CA1 and cortex regions was presented. Scale bar, 50 μm. (**b**) Percentage of cell death. For each group, at least 6 animals were included; for each animal, at least 4 brain slices were used for analysis. ^**^*P*<0.01 *vs.* Sham; ^##^*P*<0.01 *vs.* I/R.


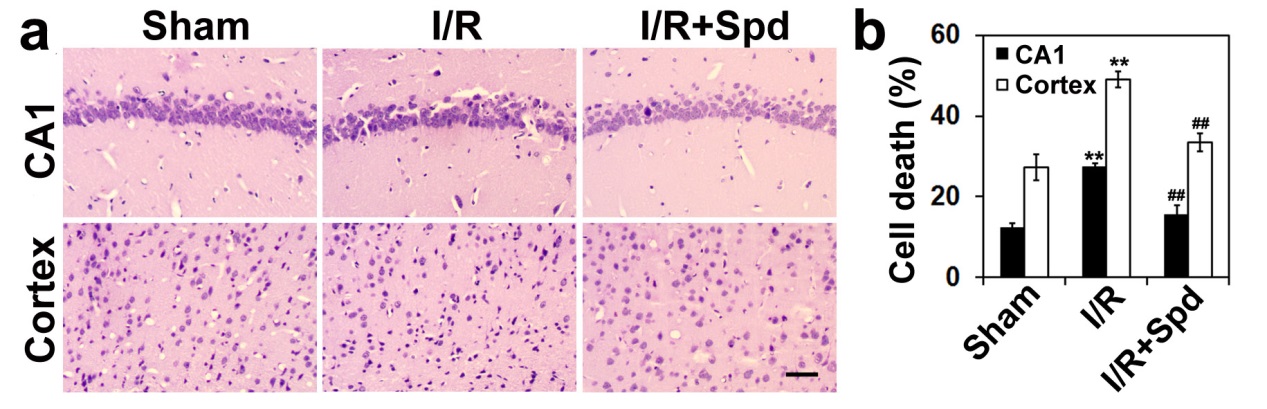


**Supplementary Table 1** Primary antibodies used in this study.

| **Antibody** | **Host species** | **Immunizing antigen** | **Dilution/Application** | **Source** | **Catalog No.** |
| --- | --- | --- | --- | --- | --- |
| Pro-Caspase-3 | Rabbit | aa 1-277 of human caspase-3 | 1 : 500 WB | Santa Cruz Biotechnology, Inc., CA, USA | sc-7148 |
| Cleaved Caspase-3 (Asp175) (5A1E) | Rabbit | N-terminal residues adjacent to Asp175 of human caspase-3 | 1 : 500 ICC  1 : 1000 IHC  1 : 500 WB | Cell Signaling Technology, Inc., Boston, MA, US | 9664 |
| Cytochrome C | Mouse | Synthetic peptide of pigeon Cytochrome C aa 1-80, 81-104 and 66-104 | 1 : 500 WB | Abcam, Cambridge, UK | ab13575 |
| LC3A/B | Rabbit | Synthetic peptide of the residues surrounding Gly40 of LC3A/B | 1 : 1000 WB  1 : 200 ICC | Cell Signaling Technology, Inc., Boston, MA, US | 4108 |
| Beclin 1 (NT) | Rabbit | 17 aa peptide near human Beclin 1 N-terminus | 1 : 1000 ICC  1 : 2000 IHC  1 : 200 IF  1 : 1000 WB | ProSci Inc., Poway, CA, US | 3613 |
| Beclin 1  (CT) | Rabbit | 16 aa peptide near human Beclin 1 C-terminus | 1 : 1000 ICC | ProSci Inc., Poway, CA, US | 3611 |
| p62 | Rabbit | Synthetic peptide of residues surrounding Gly410 of human SQSTM1/p62 protein | 1 : 1000 WB | Cell Signaling Technology, Inc., Boston, MA, US | 5114 |
| VDAC1 | Rabbit | Synthetic peptide of the residues near human VDAC1 N-terminus | 1 : 1000 WB | Abcam, Cambridge, UK | ab154856 |
| GAPDH | Rabbit |  | 1 : 200 WB | Boster, Wuhan, China | BA2913 |

aa, amino acid; ICC, immunocytochemistry; IF, immunofluorescence; IHC, immunohistochemistry; WB, western blotting.
